# Searching for a Better Animal Model for Chronic Tympanic Membrane Perforation

**DOI:** 10.3390/jpm14050513

**Published:** 2024-05-11

**Authors:** Dragoș Bularda, Roxana Șerban, Corina Butnaru, Mihai Mareș, Liviu Catalin Burtan, Luminița Rădulescu, Cristian Mârțu

**Affiliations:** 1 Department of Otorhinolaryngology, “Grigore T. Popa” University of Medicine and Pharmacy, 700115 Iași, Romaniacmbutnaru@yahoo.com (C.B.);; 2 Department of Biochemistry, “Grigore T. Popa” University of Medicine and Pharmacy, 700115 Iași, Romania; 3 Laboratory of Antimicrobial Chemotherapy, Iasi University of Life Sciences “Ion Ionescu de la Brad” (IULS), 700490 Iași, Romania; mihai.mares@iuls.ro; 4 Clinical Department, Iasi University of Life Sciences “Ion Ionescu de la Brad” (IULS), 700490 Iași, Romania

**Keywords:** animal model, tympanic membrane, chronic perforation, chinchilla

## Abstract

Chronic tympanic membrane perforation represents a prevalent otological condition, necessitating a reliable animal model for the validation and safety assessment of surgical techniques and materials employed in myringoplasty. This prospective study involved the establishment of chronic tympanic membrane perforation animal models in 16 chinchillas. A thermic myringotomy was conducted on the right ear (study group), followed by cold instrument myringotomy, coupled with the topical application of mitomycin C and dexamethasone solution on the left ear (control group). Results revealed that tympanic membrane perforations in the study group persisted for a minimum of 4 weeks in 93.7% of cases and extended to 12 weeks in 62.5% of the cases. In contrast, all tympanic membrane perforations in the control group were present at 4 weeks, with only 37.5% persisting after 12 weeks, although statistical tests did not find significant differences between the two groups (chi-square: *p*-value = 0.157, Kruskal–Wallis: *p*-value = 0.093, Mann–Whitney: *p*-value = 0.121). The thermic myringotomy employed to induce chronic tympanic membrane perforation in animals demonstrated efficiency and sustainability. This model, characterized by stability and reproducibility, holds promise for future experimental applications in the field.

## 1. Introduction

Tympanic membrane (TM) perforation is a prevalent pathology, commonly arising from various causes such as trauma, infections (otitis media), and iatrogenic factors, including tympanostomy tubes or iatrogenic events following a foreign body or cerumen removal [1]. Even in cases of necessary iatrogenic maneuvers, like tympanostomy tube insertion, the incidence of secondary perforation varies from 2.2% (for short-term tubes) to 16.6% (long-term tubes) [2].

Chronic TM perforations pose a persistent challenge in otolaryngology, requiring individualized approaches for successful resolution. The prolonged duration of non-healing introduces complexities in the underlying tissue, necessitating a comprehensive understanding of the biological and mechanical factors influencing the repair process.

This pathological condition exhibits a lack of age restriction, and the manifestation of TM perforation is possible at any life stage. The repercussions encompass not only conductive hearing loss but also an increased susceptibility to middle ear infections, the potential emergence of cholesteatoma, water sports restrictions, etc. [3]. The negative influence on lifestyle has been reported in multiple studies, underscoring the necessity for a holistic and profound comprehension of this pathological entity [2]. The multifaceted documentation in diverse scholarly investigations underlines the need for a thorough exploration of this condition, acknowledging its diverse impacts on individuals across different age groups. The profound implications on auditory health, coupled with the potential for secondary complications, necessitate comprehensive and targeted therapeutic interventions. TM perforation hinders normal behavior regarding water/showering/swimming/water sports, with the necessity of always protecting the external auditory meatus from splashing, washing, and especially diving with the head or ears in the water. Even with the utmost care and protection (including ear plugs, and cotton with cream/oil substances for water repelling), the possibility of water passing through the TM perforation exists and can give rise to complications. These complications include—otitis, ear discharge, otomastoiditis, chronic ear disease, vertigo, hearing loss, etc.

The frequency of occurrences involving TM perforation showcases substantial variability in its prevalence. Within the United States, it is estimated that approximately 1% to 3% of the population encounters instances of TM perforation at various junctures throughout their lifespans. Nevertheless, the prevalence takes on a markedly distinctive pattern in specific demographic groups, notably among indigenous Australians, where reported occurrences witness a substantial surge, with prevalence rates oscillating in the range of 28% to 43% [3]. The considerable diversity in the incidence rates underscores the multifaceted nature of TM perforation, with contextual and demographic factors playing pivotal roles in shaping the prevalence trends. Such disparities in prevalence rates prompt a deeper inquiry into the underlying determinants and contributing factors that explain the epidemiological landscape of TM perforation. A thorough exploration of these variations is essential for enhanced comprehension of the distribution patterns, paving the way for more targeted and culturally sensitive approaches to preventive measures and therapeutic interventions.

Most cases of acute TM perforation tend to undergo spontaneous healing within approximately 10 days. This natural healing process involves a complex series of molecular events, including inflammation, keratinocyte migration, fibroblastic activity, and ultimately vascular proliferation [4]. Understanding these intricate mechanisms is essential for comprehending the resilience and regenerative capacity of the TM in response to acute perforations.

A TM perforation is classified as chronic when it does not undergo spontaneous healing within three months [5]. In such cases, surgical intervention becomes necessary, along with the implementation of water precautions [6]. Notably, a substantial percentage, ranging from 6% to 20% of TM perforations resulting from trauma fail to heal properly [7]. Usually, TP perforations occur after ear tube placement, acute otitis media, chronic otitis media with or without cholesteatoma, or as a result of barotrauma [4,8].

Tympanoplasty is the surgical technique employed for repairing chronic TM perforations and middle ear reconstruction. If the procedure is limited to TM perforation repair, it is classified as type I Tympanoplasty or Myringoplasty. However, the success of tympanoplasty is contingent upon factors such as the site and size of the perforation, and etiology, but also dependent on the surgeon’s skill and experience [8].

In adults, the success rate of this intervention varies significantly, ranging between 60–99% [9]. In contrast, the success rates in pediatric cases present a controversial topic, with figures spanning from 35–94% [9,10]. Moreover, the definition of a successful tympanoplasty varies among studies. While some consider the presence of an intact TM as a successful outcome, others deem it crucial to also evaluate post-surgery hearing thresholds and the degree of middle ear aeration [11]. The diverse criteria for success underscore the complexity of evaluating outcomes in tympanoplasty procedures. In the realm of surgical interventions, tympanoplasty assumes a pivotal role in addressing chronic TM perforations. However, the success of tympanoplasty is not solely contingent on the surgical technique employed; patient-specific factors, including age, overall health, and the presence of comorbidities, contribute significantly to the outcomes. This multifactorial nature underscores the importance of tailoring treatment strategies to individual patient profiles.

Various grafting materials are utilized in the surgical repair of chronic TM perforations, with common choices including tragal cartilage, temporalis fascia, pretragal superficial musculoaponeurotic system fascia, and perichondrium [11,12,13]. Different strategies have been researched, including fibrin derivates, biomaterials, 3D printing to match the defect size, etc. with promising results [6,7,8]. Depending on the size and the duration of the perforation of the TM, different approaches can or should be used; there needs to be good access and visibility of the perforation, the anterior border being very important for good audiological results and needs to be available for inspection during the surgery and after the healing or during the follow-up. The posterior border is important for good healing after the tympanoplasty procedure and must be very carefully checked during the surgical window, in order to ensure good operating technique and cholesteatoma avoidance. Furthermore, depending on the site, the size of the TM perforation and the anatomy of the external auditory meatus, different surgical approaches may be selected—endaural, transcanal, or behind the ear. 

In recent discussions, alternative methods have been explored to facilitate TM perforation closure. Topically applied growth factors and human umbilical cord serum have emerged as potential alternatives, raising the prospect of a more straightforward approach compared to conventional tympanoplasty [14]. Moreover, the anatomical intricacies of the middle ear present unique challenges in achieving optimal results. The proximity of vital structures, such as the ossicles, the facial nerve, the round window, and sometimes the difficult anatomy (such as a high placed jugular bulb, aberrant internal carotid artery, a small antrum, etc.) demands precision in surgical maneuvers to restore auditory function while minimizing the risk of complications. Advancements in imaging technologies, such as high-resolution computed tomography (HRCT) and magnetic resonance imaging (MRI), play a pivotal role in preoperative planning, enhancing the surgeon’s ability to navigate complex anatomy with precision.

The development and enhancement of surgical techniques for chronic TM perforation repair necessitates pre-clinical testing using appropriate animal models [15]. Different animal models that are universally available have been investigated, including guinea pigs, rabbits, gerbils, pigs, etc. with various inconveniences (small TM, difficult visualization and manipulation, tortoise external ear canal, limited availability of specimens) [4]. Recognizing the high incidence of chronic perforation of the TM and the absence of consensus on the optimal surgical techniques and materials, our study aims to establish a stable and reproducible model of chronic TM perforation, specifically in chinchillas. 

The objective is to contribute valuable insights into the selection of appropriate animal models for future research, allowing for advancements in both understanding and addressing this prevalent condition. Chronic TM perforation remains a complex and multifaceted challenge, demanding an integrative and innovative approach. Through a confluence of advanced surgical techniques, regenerative medicine, and collaborative research endeavors, the medical community stands poised to redefine the landscape of chronic TM perforation management, ultimately improving patient outcomes and quality of life.

## 2. Materials and Methods

The primary objective of this study was to validate and replicate a chronic TM perforation on an animal model. The prospective investigation was conducted on adult male chinchillas, involving a total of sixteen subjects with weights ranging from 300 to 650 g.

### 2.1. Study and Control Groups

The chinchillas were divided into two distinct groups: a control group (represented by the left ears), and a study group, where the right ears were the focus of observation and experimentation.

To establish chronic TM perforation in the control group, the methodology outlined by Wang in 2015 was employed [15]. This standardized technique was chosen for its documented efficacy and relevance to the study’s objectives. The controlled application of this perforation method allowed for a consistent and reproducible approach to creating TM perforations in the designated ears of the chinchillas.

### 2.2. Methodology

Before starting the experiment, each chinchilla’s ears were thoroughly examined using a Zeiss microscope and a Hopkins oto-telescope to facilitate precise observations and to rule out any middle ear diseases. The debris from each external auditory canal was removed using a suction tube. Also, the ear canals and the surrounding skin were cleaned with an iodine solution, so the myringotomies could be performed under aseptic conditions.

The induction of general anesthesia in the chinchillas involved an intramuscular injection administered between the semimembranosus and semitendinosus muscles. The anesthetic agents utilized were ketamine at a dosage of 40 mg/kg and xylazine at a dosage of 2 mg/kg [16]. 

In the study group, a subtotal thermal perforation was performed. This involved using the tip of a stainless-steel port cotton, heated in a flame until achieving a light cherry red color (measured between 290–310 °C with a thermocouple). The carefully controlled thermal perforation, executed in the TM of the study group, provides a standardized approach for the study. 

In the control group, a simple myringotomy was performed via transcanal, using a Wullstein needle. For the topical application, a combined solution of 2 mL was prepared, containing Dexamethasone (DXM) at a concentration of 4 mg/mL and Mitomycin C (MMC) at a concentration of 0.4 mg/mL, both in equal proportion. Using a soaked gel foam, the solution was applied to each left ear of the 16 chinchillas—immediately after the myringotomies. After a waiting period of 10 min [15], the gel foam was removed.

### 2.3. Follow-Up

The assessment of TM perforation status was conducted under general anesthesia, with initial evaluations performed after 2 weeks and subsequent monthly assessments over a three-month period. TM perforations were categorized as either completely closed or open during these follow-up sessions.

To execute the TM perforation and subsequent examinations, a Zeiss surgical oto-microscope was utilized. For monitoring the healing process, a Hopkins oto-telescope with a 0° diameter, measuring 1.9 mm in diameter and 10 cm in length, was employed.

### 2.4. Statistic Testing

Statistical analysis involved comparing the closed and open TM perforations for each ear at specified time intervals. The results were expressed as percentages and the statistical significance of the final outcomes was determined using the chi-square test.

It is important to note that the study adhered to ethical standards and received approval from the local ethics committee. The research was conducted in strict accordance with ethical principles and legislation governing the protection of animals used in research [17]. This underscores the commitment to ensuring the ethical conduct of the study and the welfare of the involved animals.

## 3. Results

Following a comprehensive 12-week observation period, noteworthy findings emerged, indicating that 50% (16 out of 32) of the TM in the study group remained open, as detailed in Table 1. On a global scale, the data highlighted that 96.875% (31 out of 32) of the TM perforations were still open at the conclusion of week 4, with a subsequent reduction to 65.625% (21 out of 32) by week 8, revealing the evolving nature of the perforations (Table 1).

**Figure 1:**
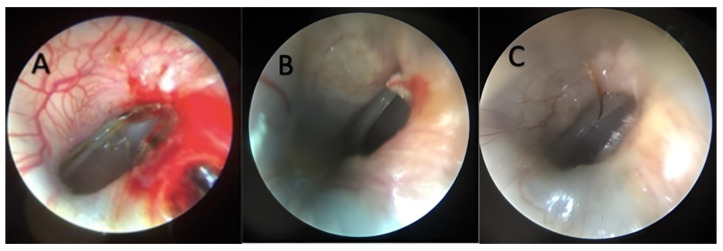
(**A**–**C**) Three different tympanic membranes from the study group, 12 weeks after the myringotomy.

Within the study group, a notable trend was observed: at the 4-week mark, one TM perforation had successfully closed and this positive trajectory continued, culminating in the closure of 6 TM perforations after the 12-week period, as systematically documented in Table 2. However, the persistence of TM perforations was still evident in 62.5% (10 out of 16) of the study group after a substantial 3-month duration, as visually represented in Figure 1.

**Figure 2:**
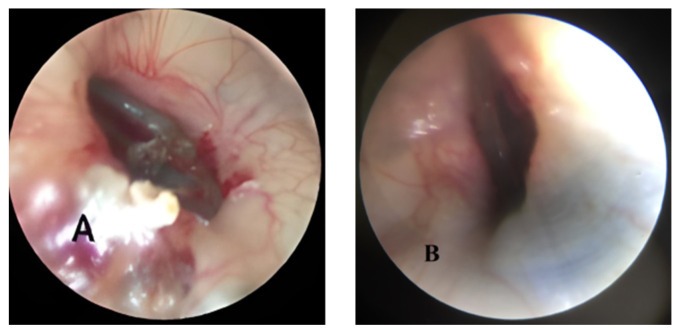
(**A**,**B**) Open tympanic membranes in the control group after 12 weeks.

In contrast, the control group exhibited a distinctive pattern—no complete closures were noted after the initial 4 weeks; at the 8-week milestone, 11 TM perforations were still present, constituting 68.75%. At the 12-week follow-up, a reasonable proportion of TM perforations, accounting for 37.5% or 6 ears, remained open, as shown in Figure 2.

Conducting the statistical analysis, as presented in Table 3, no significant differences between the study and control groups have been observed, the *p*-value is 0.157299 (not significant at *p* < 0.05). It is noteworthy that throughout the entire study duration, no instances of ear infections occurred, and none of the chinchillas were sacrificed, affirming the overall well-being and ethical conduct of the study subjects.

## 4. Discussions

The chinchilla is an animal model more usually used for otological research because has a wide external auditory meatus that assures a convenient surgical approach to the large TM and also because the middle ear cavity and the Eustachian tube have a close resemblance with human ear anatomy.

The attempt to create an animal model dates from 1992, the reviews in the literature offer many studies, that present the experience of different authors in developing a stable, reproducible model and simple to produce. Amoils et al. [18] obtained a chronic TM perforation in 32 ears (84% cases) at 6 to 8 weeks after thermal perforation in chinchillas. Their study was different from ours in many aspects. The most important is the fact that they did not specify the temperature at which they performed the perforations, or this is the most important point in the experiment. 

All ear surgeons know that perforations secondary to welding sparkles are the most difficult to close because the damages resulting from the high temperature (more than 1000 °C in these cases) are extremely severe; the higher the temperature, the more difficult to close a TM perforation. Spontaneous healing in these situations is almost impossible [19,20,21]. The authors prescribed systemic antibiotics for 5 days. Usually, the antibiotics are given after an accidental perforation of the TM to aid the healing process and not to facilitate the persistence of the perforation. Also, they folded the edges of the perforation, and last, but not least, the healing process was followed up just for 6–8 weeks. In our study we used a perforator heated at 300° Celsius, we did not use antibiotics at all (and we did not encounter any infection), and we did not fold the borders of the perforation. 

In the investigation conducted by Wang and colleagues in 2016 [22], focusing on murine subjects, an examination of the factors influencing the development of a chronic TM perforation revealed an intricate association with the rates of epidermal growth, collagen and keratin deposition, and the reduction in cell proliferation. Notably, within a span of 3–4 weeks, Wang observed a discernible transition of the TM perforation from an acute state to a chronic one. The experimental protocol in this study involved the application of topical mitomycin C (MMC) and dexamethasone (DXM) in tandem with ventilation tubes, leading to a persistent perforation rate of 70% at the conclusion of the 10-week observation period [22]. Another study reports 100% patency of TM perforation after 6 weeks after using the same solution for 3 consecutive days but with some showing signs of healing and some of infection [23].

The technique proposed by us is simpler, faster, with less medication and manipulation, with the persistence of the perforation for longer than 8 weeks, up to the point of 12 weeks (10 out of 16–62.5%), when one can discuss of chronic TM perforation.

Despite the discussed variations, our methodology stands out for its cost-effectiveness and simplicity when compared to Wang’s elaborate technique. Although the statistical analysis demonstrated no significant differences between the two methodologies, the comparable efficacies and supporting the viability of the thermic perforation technique as a pragmatic alternative in murine models of chronic TM perforation are noteworthy.

Another study in which MMC and DXM are used is the one performed by Langston et al. on mice [2]. In their study, fractional radiation with Cesium-137, topical application of lipopolysaccharides, and a combined solution of DXM and MMC were used. The application of DXM/MC reliably produced TM perforation that lasted at least 8 weeks in 86.48% of cases. The rate of persistent perforation at 8 weeks is better than ours (68.75%) but without being statistically significant, and it gives no information regarding the behavior of the perforation at 12 weeks.

In 2019 Esteban et al. performed a study of the patency of TM perforation and the influence of different agents in creating a chronic TM perforation [24]. In one of their study groups (in the successful group)—they used MMC in the same concentration as we did and the results at 8 weeks were comparable with ours being 62.5% of open TM vs 68.75% open TM in our study.

As Jassir et al. found, the duration of the persistence of the perforations of TM is related to the MMC concentration [25]. This can explain almost the same rate of perforation closure of the TM at 8 weeks. In our study group, in the thermic perforation group, the rate of persistent perforation at 8 weeks was 62.5% being equal to Esteban’s rate for the MMC/DXM group, again with incomparably low costs. They did not follow up longer on the evolution of perforations, while our study group remained stable and the control group suffered further closure of perforations up to the 12-week follow-up.

Truy employed a solution of glutaraldehyde at a concentration of 2% in a meticulous endeavor to induce a chronic perforation pattern in a comprehensive study involving dogs. The investigative team achieved notable outcomes, as evidenced by the acquisition of six persistent perforations out of a total of 14 cases meticulously monitored over a substantial 15-week duration, as extensively documented in their comprehensive study [26]. The implementation of glutaraldehyde at the specified concentration emerged as a key factor in the experimental design, elucidating its role in the successful induction of a chronic perforation pattern within the canine subjects under scrutiny. The noteworthy success rate and the protracted observation period further contribute to the depth of insights gleaned from this research, underscoring the intricate dynamics involved in the formation and persistence of chronic TM perforations in canine models.

In their work performed on 8 chinchillas, Emami et al. conclude that thermal tympanotomy in combination with infolding technique is not a reliable and consistent method to create a chronic perforation of the tympanic membrane, the closing time being very short (4–6 weeks), again the paper is not telling us the temperature used to perform the perforations [27]. In our case, at 300 °C we obtained a persistent TM perforation in 10 out of 16 ears at 8 weeks in the study group.

Thermal perforation was also used by Wieland in his experiment on chinchilla model [28]. To prevent spontaneous closure of the perforation, prednisolone or MMC was applied topically directly to the tympanic membrane. The chronic TM perforation model was also tried by LASER and compared with the infold technique and infold combined with tube insertion with moderate results [29].

In our study, we performed a simple and low-cost technique—thermal myringotomy—and compared it with an expensive model (MMC/DXM). After a follow-up of 12 weeks, the thermal perforations were patent in 62.5% of cases, compared to 37.5% in the MMC/DXM group. Although the difference was not statistically significant (*p* = 0.157299 at *p* < 0.05) (Table 3), the data support the thesis that thermal myringotomy is better than simple myringotomy and MMC/DXM application. In spite of the meticulous design of the study, providing a robust foundation for the interpretation of results, further expansion of the groups and observation milestones at a higher timespan might bring additional statistical meaning into the proposed model.

Whilst two additional tests generally used for investigating differences between small samples were also performed, we did not obtain evidence of significant differences between the study and control group. First, the Kruskal–Wallis test did not reject the null that the population medians of the two groups are equal (*p*-value = 0.093). Furthermore, the Mann–Whitney test could also not reject the null claiming that the median or the distribution underlying the study sample is the same as the ones in the control sample (*p*-value = 0.121) (see Table 4).

The present study has some limitations represented by the inability to realize a perforation of standard dimensions, in the study group the TM literally dissolved when we touched it with the cherry red heated stainless-steel port-cotton. The size of the perforation can influence the upcoming therapeutic decision and the surgical technique used to close the defect. We think that raising the temperature a little more would result in a more successful model, but it is important to find a balance because, beyond a certain temperature, the damage of the tissue will become inappropriate for the purpose of the model—namely to study new surgical techniques and new materials for tympanoplasties. Also, the size of the tip of the heated instrument might control the size of the perforation.

## 5. Conclusions

The text highlights the development and comparison of two TM perforation models in chinchillas. The study introduces a novel and more accessible thermal myringotomy model, contrasting it with a more expensive model involving the use of Mitomycin C (MMC) and Dexamethasone (DXM).

The choice of chinchillas as an animal model for otological research is justified by their anatomical features, including a wide external auditory meatus and close resemblance of the middle ear cavity and Eustachian tube to human ear anatomy. We discussed the previous attempts to create a stable, reproducible model for chronic TM perforation in chinchillas, emphasizing the limitations of existing models, including incomplete details on the temperature used during thermal perforations and concerns about tissue damage.

The study introduces a simplified thermal myringotomy model, using a perforator heated at 300° Celsius. This model avoids the use of antibiotics and other drugs that can be expensive or difficult to obtain, with a variable rate of individual reactivity, does not involve folding the borders of the perforation, and has a follow-up period of 12 weeks. The thermal myringotomy model is compared with a more complex model involving the topical application of MMC and DXM. Despite variations, the thermal model achieves a persistent TM perforation rate of 62.5% (10 out of 16) at 12 weeks, comparable to the MMC/DXM model’s 37.5% (6 out of 16), although the difference was not statistically significant (*p* = 0.157299 at *p* < 0.05).

The thermal myringotomy model is emphasized for its cost-effectiveness, efficiency, and simplicity compared to the MMC/DXM model. The study suggests that the thermal model may be more favorable for studying new surgical techniques and materials for tympanoplasties. While the study acknowledges limitations in achieving standard perforation dimensions, it suggests that further optimization of the thermal myringotomy model may enhance its success without compromising tissue integrity.

The study introduces a promising alternative for a chronic TM perforation model in chinchillas, emphasizing its simplicity, cost-effectiveness, and potential comparability to more complex models. The findings open avenues for further research in optimizing the model and exploring its utility in studying tympanoplasties.

## Figures and Tables

**Table 1:** Summary of closed versus opened TM aspects for all the animals.

TM Perforation	Day 0	Week 2	Week 4	Week 8	Week 12
Closed	0	0	1	11	16
Opened	32	32	31	21	16

**Table 2:** Summary of opened TM perforation evolution over 12 weeks for the two groups.

TM Condition	Day 0		Week 2		Week 4		Week 8		Week 12	
	Study Group	Control Group	Study Group	Control Group	Study Group	Control Group	Study Group	Control Group	Study Group	Control Group
Open TM	16	16	16	16	15	16	10	11	10	6
Closed TM	0	0	0	0	1	0	6	5	6	10

**Table 3:** Chi-square test applied to the two groups with 10 perforations at w12 in the study group and 6 perforations in the control group. The chi-square statistic is 2. The *p*-value is 0.157299 (not significant at *p* < 0.05).

Groups	Perforated TM	Closed TM	Marginal Row Totals
Study group	10 (8) [0.5]	6 (8) [0.5]	16
Control group	6 (8) [0.5]	10 (8) [0.5]	16
Marginal column totals	16	16	32 (Grand Total)

**Table 4:** Non-parametric tests of differences between TM closing duration in the two samples.

Test	Result
Kruskal-Wallis H test for independent samples	Statistic = 2.817, *p*-value = 0.093
Mann-Whitney U test for independent samples	Statistic = 6.000, *p*-value = 0.121

Note: The significance level was established at 5%. The tests included the observations with closed perforation in both study and control until week 12 (N = 10, 5 in study group and 5 in control group). The statistics are corrected for ties.

## Data Availability

Data is contained within the article.
